# Treatment of Idiopathic Parkinson's Disease with Traditional Chinese Herbal Medicine: A Randomized Placebo-Controlled Pilot Clinical Study

**DOI:** 10.1093/ecam/nep116

**Published:** 2011-06-18

**Authors:** Wan Fung Kum, Siva Sundara Kumar Durairajan, Zhao Xiang Bian, Sui Cheung Man, Yuen Chi Lam, Li Xia Xie, Jia Hong Lu, Yan Wang, Xian Zhang Huang, Min Li

**Affiliations:** School of Chinese Medicine, Hong Kong Baptist University, Kowloon Tong, Hong Kong

## Abstract

The objective of this clinical study is to examine the effects of a Chinese herbal medicine formula (*Jia Wei Liu Jun Zi Tang: JWLJZT*) on motor and non-motor symptoms, and on complications of conventional therapy in idiopathic Parkinson's disease (PD), using an add-on design. Fifty-five patients with PD were randomly allocated to receive either Chinese herbal medicine or placebo for 24 weeks. Primary outcome measure was the 39-item Parkinson's Disease Questionnaire (PDQ-39). Secondary outcome measures included the Unified Parkinson's Disease Rating Scale (UPDRS), Short-Form-36 Health Survey (SF-36), Geriatric Depression Scale (GDS), home diaries, and a range of category rating scales. *JWLJZT* resulted in a significant improvement in the UPDRS IVC when compared with placebo at 12 weeks (*P* = .039) and 24 weeks (*P* = .034). In addition, patients in the Chinese herbal medicine group also showed significant improvement in PDQ-39 communication scores at 12 weeks (*P* = .024) and 24 weeks (*P* = .047) when compared with the placebo group. There were no significant differences between treatment and control groups for SF-36 variables, GDS score or the mean daily “on-off” time. One case of mild diarrhea was noted in the treatment group. The findings suggest that *JWLJZT* can relieve some non-motor complications of conventional therapy and improve the communication ability in patients with PD. The results of this pilot study warrant larger multi-center clinical studies to assess long-term efficacy and tolerability of *JWLJZT*, and to elucidate the mechanisms by which it affects PD function.

## 1. Introduction

Parkinson's disease (PD) is a neurodegenerative disease with prominent motor impairments that include resting tremor, rigidity, bradykinesia and postural abnormality. Epidemiological studies show that the prevalence of PD in industrialized countries is usually estimated at 0.3% of the whole population and at *∼*1% in people over 60 years of age [1]. A previous study of the Chinese population in Hong Kong reported that the prevalence among those aged ≥55 years was 0.5% [2].

Despite various advances in the understanding of PD, pharmacological treatment of PD by Western medicine is mainly for symptom management. Among different pharmacological treatments, levodopa remains the most efficacious and is still the mainstay of therapy [3]. However, long-term use of levodopa can cause disabling motor complications, particularly dyskinesias and motor fluctuations, which limit its usefulness [4]. Both of these motor complications have been found to occur in *∼*40 and 70% of patients after 5 years and 15 years of levodopa treatment, respectively, [5, 6]. Moreover, combined with motor abnormality, many non-motor aspects of PD can significantly affect patients' quality of life by causing such problems as autonomic dysfunction, constipation, nausea, sleep disruption, pain, excessive daytime sleepiness and mood disturbances. These problems often do not respond to and may even be worsened by conventional medical treatments.

In view of the undesirable long-term side effects of Western medicine, many patients seek alternative treatment for PD. Traditional Chinese medicine (TCM) has been used for centuries to treat conditions such as trembling of hands and shaking of head that correspond to the modern term “PD”. Up to the present, Chinese herbal medicine remains very popular for management of PD in Asian countries such as China, Korea and Japan. In a study done by Rajendran et al., it was observed that 40% of patients with PD use at least one form of alternative therapy [7]. Herbal medicine is one of the three most popular forms of alternative therapy adopted by PD patients. However, there are very few rigorously designed clinical trials that examine the efficacy of TCM in PD [8]. One study from Japan evaluated the role of Chinese herbal medicine in patients with antipsychotic-induced Parkinsonism [9]. With the use of a standard 10-herb formula, the investigators demonstrated a significant reduction in tremor in patients.

Unlike Western medicine, TCM diagnoses of PD fall into different categories according to the patient's fundamental constitution. According to TCM theory, PD is a condition that represents a depletion of energy, especially in the spleen and stomach. Herbal drugs have therefore been used in the treatment of PD under the general guideline of “strengthening the spleen and regulating the stomach”. “*Jia Wei Liu Jun Zi Tang*” *(JWLJZT)* is an ancient formulation developed by a TCM doctor *Zhang Lu* in 1695 AD, with the specific function of tonifying the energy (*Qi*) of spleen and stomach; it has been used to treat symptoms that are now defined as PD [10]. In this study, we determined the effect of this formulation of Chinese herbal medicine on the symptoms and quality of life of patients with idiopathic PD.

## 2. Methods

### 2.1. Patients

All idiopathic PD patients (aged 20–80 years) who presented to the Mr and Mrs Chan Hon Yin Modern Chinese Medicine Research and Service Centre of the Queen Elizabeth Hospital, Hong Kong, were screened for eligibility. Diagnosis of idiopathic PD was based on UK Brain Bank criteria [11]. Inclusion criteria were Hoehn & Yahr (H&Y) stage <5, optimal and stable levodopa treatment for at least 1 month prior to beginning the study, presence of motor fluctuations and dyskinesias during the center visits, and stable medical condition that would enable the participant to finish the treatment protocol. Even when a patient's medical condition needed adaptation, that is, due to occurrence of other mild illnesses but not severe concurrent illness, such as active cardiac, liver, renal or neoplastic disease, this adaptation did not lead to exclusion.

Patients with atypical or secondary Parkinsonism, pronounced clinically relevant cognitive impairment (Mini Mental State Examination score <24), severe concurrent illness or history of psychotic illness were excluded. Women who were pregnant, lactating or not maintaining proper contraception were not eligible. Concomitant use of Chinese medicines other than the study drug was not allowed. After assessment by a neurologist, eligible patients were assessed by a TCM practitioner to ensure their eligibility for the current TCM formulation according to TCM theories. Patients had to be diagnosed with the TCM syndrome of “asthenia of the spleen and stomach” before randomization [12]. Informed written consent was obtained from all eligible patients, and the study protocol was approved by the Ethics Committee of the Hong Kong Baptist University's Institutional Review Board (approval code: HASC/05-06/12).

### 2.2. Trial Design

This was a prospective, randomized double-blinded placebo-controlled study. Patients were randomly allocated to receive either 24 weeks of active herbal formulation or placebo with each dose of their levodopa treatment. Stratified blocked randomization was carried out by dividing the sample at baseline into participants with H&Y stages I, II, III or IV, and then carrying out a blocked randomization within each of these four strata by a computer-generated list of random numbers. Randomization information was kept in a blinded format by an independent research assistant (S.C.M.) who had provided the active drugs and placebos. It was kept in a location far away from the clinic where patients were being assessed. Emergency envelopes with the randomization code were also kept with the TCM practitioner (Y.C.L.) at the School of Chinese Medicine in Hong Kong Baptist University. The assessor (W.F.K.) and patients were blinded to treatment allocation for the duration of the study. Treatment assignments were not revealed until the study was finished. The study started in February 2007 and finished in June 2008.

### 2.3. Herbal Preparation

For this study, we used herbal granules prepared according to the formula of TCM doctor *Zhang Lu* of the *Qing* Dynasty (1695). This formula is specific for the TCM syndrome that matches symptoms of PD, and it is the basis of many over-the-counter patent medicines that are widely available in Chinese herbal stores throughout the world. Based on the original recipe for *JWLJZT*, the herbal preparation used in this study contained 11 herbs as listed in Table 1. The production of herbal granules was done by the GMP plant of Eu Yan Sang Ltd (Hong Kong). Extraction was done in a single batch to ensure consistent quality. A mixture of the herbs was extracted with hot water; after that, the aqueous extract was concentrated, spray-dried and put in sealed opaque aluminum bags. All herbs were obtained from qualified suppliers in Hong Kong and authenticated at the Chinese Manufacturers Association Testing and Certification Laboratories and the Eu Yan Sang Research and Analysis Laboratory on the basis of standards specified in the Pharmacopoeia of the People's Republic of China (2005 edition). These tests included macroscopic and microscopic examination of cross-sections and powders, chemical tests, and/or chromatographic analyses. Contamination screening for heavy metals, pesticides, and a microbial limit test were performed to ensure safety for human utilization. A placebo of similar appearance, size and taste was made using lactose, citric acid, caramel and *Momordica charantia* fruits. This was packed in the same manner as the active herbal medicine in opaque aluminum bags. All packages were distributed by an independent researcher in another room after the patients' visits. Patients were instructed to dissolve the granules in each package in hot water and drink once daily.

### 2.4. Assessments

Baseline evaluation was performed before randomization and comprised: the 39-item Parkinson's Disease Questionnaire (PDQ-39 including all dimensions and the single index) [13]; Unified PD Rating Scale (UPDRS) [14]; Short Form-36 Health Survey (SF-36) [15]; Geriatric Depression Scale (GDS) [16]; and Deficiency of Spenic *Qi* Scale (DSQS) [12]. Side effects from conventional treatment, for example, mouth dryness, constipation, nausea, insomnia, palpitation, on-off phenomenon, dyskinesias and mental problems, were assessed in a structured interview developed for this purpose. The home diary was completed by the patients or their care-givers covering the period from 6 AM till midnight (divided into 30-min intervals) for the 3 days preceding the subsequent visit. For each half-hour segment, it was recorded whether the patient was “on”, “off” or “in bed”. The proportion of “off” time while awake was calculated by dividing the total number of “off” hours over 3 days by the total number of hours awake. Formal instruction for the patients to complete the home diary was given during the first visit. Compliance was defined by the use of >70% of study herbal medicine, and was determined by counting the returned TCM packages. Adverse events (AEs) were reported spontaneously by the patient or observed by the investigator; the severity, outcome and hypothetical cause of each reported AE were assessed and recorded.

The same TCM practitioner (W.F.K.) also assessed the patients during each visit based on standard TCM practice, including inspection, auscultation and olfaction, interrogation, pulse-taking and palpation. Alteration of the study herbal medications was not allowed during the study period.

Follow-up evaluations were held for all patients at 12 weeks (on treatment) and 24 weeks (end of treatment). Assessment was carried out in the “on” state, that is, time of optimal medication effect as defined by the patient. Follow-up examinations were carried out at similar times of the day to attenuate effects of motor fluctuations.

### 2.5. Statistical Analysis

Based on previously published studies [17, 18], a sample size of 36 for the study was calculated to be sufficient to enable detection of a reduction in the primary outcome measure (PDQ-39 single index) by 11 points with 80% confidence to a significance level of 0.05.

All efficacy analyses were performed in the intent-to-treat (ITT) population, which comprised all randomized patients who had a baseline measurement and at least one measurement while on the study drug. Statistical analysis was performed using SPSS software version 12 (SPSS, Chicago, IL). Normality of data was checked by K-S test. For normally distributed data, independent Student's *t*-test was applied to 12 weeks and 24 weeks—baseline differences, respectively, for the two groups of patients. Differences within groups before and after the 12 and 24 weeks treatment were determined using paired *t*-test. For non-normally distributed data, the Mann-Whitney U-test or Wilcoxon's rank sum test was used as appropriate. A *P*-value ≤.05 was considered to be statistically significant.

## 3. Results

### 3.1. Patients

**Figure 1:**
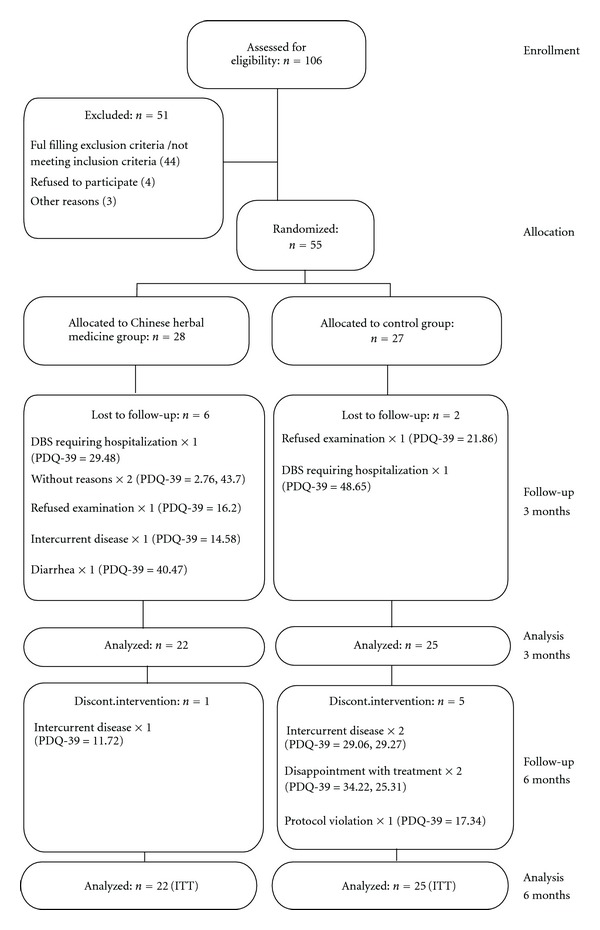
Patients flow. Dropouts are given along with their PDQ-39 summary index score at baseline.

A total of 106 patients with PD were screened, of which 55 met the inclusive criteria and were randomized. Twenty-eight patients were allocated to receive herbal medicine and 27 to receive placebo. The patient flow and reasons for exclusion are shown in Figure 1. Eight subjects were excluded from the analysis because they left the study before any post-baseline testing. Analysis was performed on the remaining 47 subjects, 22 in the TCM group and 25 in the placebo group. There were no significant differences between the two treatment groups with regard to baseline characteristics (Table 2). A total of 14 (25.5%) patients withdrew from the trial during the study period: 7 (25%) in the TCM group and 7 (25.9%) in the placebo group. Twenty-one patients in the TCM group and 20 patients in the placebo group completed the study.

### 3.2. Treatment Effects within Groups

The mean score of PDQ-39 single index, total UPDRS and other parameters with significant change are shown in Table 3. TCM therapy significantly reduced the complications of therapy from 4.22 points at baseline to 3.36 points at 12 weeks and 3.22 points at 24 weeks (UPDRS Part IV; *P* < .05). UPDRS Part IVC (other complications) scores in the TCM group were significantly improved from 1.09 to 0.64 points and 0.50 points at 12 and 24 weeks, respectively, (*P* < .05). Communications at 12 and 24 weeks significantly worsened in the placebo group from baseline 23.75 to 30.67 points and 29.67 points, respectively, (PDQ-39 Communications scale; *P* < .05). There was no difference between control group and TCM group in the items of the SF-36 Health Survey. There were also no significant changes in other parameters including GDS, DSQS, mean “off” time, and the structured interview items in the test and the control groups (Data not shown).

### 3.3. Group Differences

Comparisons between the TCM group and the control group in PDQ-39 single index, total UPDRS and other parameters with significant difference are summarized in Table 4. The PDQ-39 Communications score improved in the TCM group but worsened in the control group. Group differences for this scale were significant at both time points (*P* < .05). There was no group effect on the rest of the sections of PDQ-39. TCM reduced complications of therapy (UPDRS Part IV) more than placebo at 12 weeks (*P* = .03) but not at 24 weeks. However, there was a trend for TCM to improve the UPDRS Part IV score more than placebo at 24 weeks (*P* = .09). UPDRS Part IVC (other complications) score improved significantly more with TCM than with placebo at both time points (*P* < .05). There was no group effect on other parts of UPDRS (data not shown). For the measures in SF-36, GDS, DSQS, mean “off” time, and the structured interview items, there were no significant differences between the TCM group and the placebo group (data not shown).

### 3.4. Adverse Events

Most patients tolerated the study drug well. One patient in the TCM group suffered from mild diarrhea. No other adverse effects were reported by patients.

## 4. Discussion

Despite widespread enthusiasm for TCM among the public, there is little controlled clinical data on its efficacy. In this research, we studied the efficacy of an ancient TCM formulation in treating idiopathic PD in Chinese patients. This study has various special features. First, while there are significant differences in the theory between TCM and conventional Western medicine, we were able to include patients with a diagnosis confirmed by both Western criteria of PD (UK Brain Bank) and TCM diagnostic criteria [11, 12]. This design ensured the inclusion of patients that would potentially benefit from this herbal medicine. Next, different from many previous studies on TCM treatment of PD [19], this was a randomized double-blinded placebo-controlled trial. All herbal medicine and placebos were packaged in identical aluminum bags to assure satisfactory blinding of the patients and assessors. Moreover, we assessed each patient's health-related quality of life in addition to his/her PD symptoms.

The results of this study showed that *JWLJZT*, made from a Chinese herbal decoction, significantly reduced some non-motor complications in patients with PD under conventional medicine treatment. The major improvement was reflected by the reduction of score in UPDRS IVC which assessed gastrointestinal disorders, such as anorexia, nausea and vomiting, sleep disturbances, such as insomnia and hypersomnolence, and symptomatic orthostasis. In addition to the UPDRS IVC results, our results showed significant improvement in the whole UPDRS IV in *JWLJZT* groups after 12 and 24 weeks of treatment. However, these results are not conclusive because analysis of the UPDRS IV as three separate parts showed no significant changes in the dyskinesias section (UPDRS IVA) nor in the clinical fluctuations section (UPDRS IVB). Also, there was no significant change in the mean daily “off” time in both groups as measured by home diaries, despite a trend of reduction of mean daily “off” time in *JWLJZT* groups at 24 weeks (baseline: 16.65  ±  17.59 versus 24 weeks: 13.15 ± 16.58, *P* = .097). Our results are supported by the findings of a previous clinical trial which assessed the efficacy of another herbal medicine, *“Zhenzhanning”(ZZN)*, in PD [20]. It was reported that frequency of nausea, vomiting, anorexia and orthostatic hypotension of the test group decreased significantly more than in the control group. In addition to this latter study, other clinical experiments with Chinese herbal medicines have demonstrated significant improvement in non-motor complications of conventional therapy, including reduced sleeplessness, vivid dreaming, constipation, dizziness and fatigue [21–23].

A previous clinical study evaluated the effects of a prescription similar to ours (six of the herbs present in our formulation are also in their formulation) [24]. Hiyama et al. assessed the efficacy of *Liu Jun Zi Tang* (*LJZT*) in treating PD patients with clinical fluctuation. It focused on the influence of reduced gastric emptying in PD patients which may lead to fluctuations in the plasma levels of levodopa. As a result, they used *LJZT* and levodopa to treat seven PD patients with motor fluctuations. Assessment included observation of tremor, rigidity and bradykinesias, the patient's subjective scoring scale, absorption of Acetaminophen, and plasma level of levodopa. Two weeks later, the results showed that *LJZT* significantly improved patient's mobility and percentage of mean daily “on” time. Moreover, frequency of gastric discomfort, anorexia and nausea were lowered. In the meanwhile, improvement in gastric emptying and stability of plasma levodopa level was noted. This may explain why, in our study, *JWLJZT* could alleviate non-motor complications that are acute side effects of levodopa such as nausea, vomiting and orthostatic hypotension because these effects have been shown to be correlated with plasma levodopa concentrations [25]. Further basic research is required to explore the underlying mechanisms in detail. Our study was designed simply to verify the effects, not to elucidate the mechanisms of these effects of *JWLJZT*.

Another important observation of our RCT is that *JWLJZT* significantly affects communications as assessed by the PDQ-39. Throughout the study, the control group's communication scores worsened, while in the treatment group, improvement was noted at 12 and 24 weeks. Differences between these two groups were significant. PDQ-39 measures communication in different contexts, including difficulty with speech, feeling unable to communicate properly and feeling ignored by people. In fact, changes in speech and voice in PD patients occur in up to 80–90% of cases and these changes can affect intelligibility, ability and desire to communicate [26, 27]. In general, individuals feel they have lost control in communication, are less confident and have difficulty getting their message across, with subsequent frustration, feelings of inadequacy and feelings of loss of independence [28]. In consequence, social isolation may occur in those patients which greatly reduces their quality of life and leads to depression [29]. Our findings thus provide a new therapeutic approach for those PD patients with communication difficulties. Before our study, Cao and colleague assessed the effects of a Chinese herbal medicine “*Naokangtai* capsule” on 62 cases of PD patients [30]. Their results showed that patients improved in communications after 3 months of TCM treatment. Nevertheless, we cannot exclude the possibility that TCM doctor consultation and attention may have an impact on these outcomes in our test group. Such effects have never been studied. Moreover, communication is sensitive to a variety of life stressors. Thus, it is hard to discriminate the treatment effects from those confounding factors. Therefore, the effects on communications observed in our study should contribute to the ongoing discussion on possible mechanisms of action and surely deserve consideration in further studies. For example, further clinical studies using the specific “Self-Perceived Communication Competence Scale” (SPCC) would be able to evaluate the effect of TCM on communication in depth [31].

In this study, *JWLJZT* had no effect on Parkinsonian motor abnormality. Distinct from conventional Western medicine, individualized treatment according to patient's current TCM symptom complex is regarded as a major feature of TCM. Nevertheless, because of difficulties in standardization and sufficient blinding for individualized treatment, we have not assessed the efficacy of individualized treatment in this trial. The potential drawback of standardized treatment is a possible loss in the efficacy of the Chinese herbal medicine treatments due to a lack of individualized therapy, as performed in the traditional way. A clinical trial with proper design is merited to evaluate the efficacy of individualized herbal treatment in patients with PD [32].

In conclusion, the 11-herb TCM prescription resulted in significant improvement in some non-motor complications of conventional medicine treatment and communications abilities in patients with idiopathic PD. Studies of the biochemical mechanisms by which *JWLJZT* affects PD could point the way to further advances and even better clinical treatment of PD. Also, studies with larger sample sizes are required which would have increased statistical power and would be able to better determine if *JWLJZT* can offer long-term control of PD and retard the progression of this disease.

## Funding

Grant EYS/07-08/01 from Eu Yan Sang (Hong Kong) Limited; faculty research grants FRG/08-09/I-01 from Hong Kong Baptist University.

## Figures and Tables

**Table 1:** Herbal preparation

Chinese name	Pharmaceutical name	Percentage
*Dang shen*	Dried root of *Codonopsis pilosula (Franch.)* Nannf. (Fam. Campanulaceae)	13.39
*Sheng di*	Dried root tuber of *Rehmannia glutinosa* Libosch. (Fam. Scrophulariaceae)	13.39
*Fu ling*	Dried sclerotium of the fungus, *Poria cocos (Schw.)* Wolf. (Fam. Polyporaceae)	10.71
*Gou teng*	Dried hook-bearing stem branch of *Uncaria rhynchophylla (Miq.)* Jacks. (Fam. Rubiaceae)	10.71
*Bai Zhu*	Rhizome of *Atractylodes macrocephala* Koidz. (Fam. Compositae)	8.93
*Dang gui*	Dried root of *Angelica sinensis (Oliv)* Diels. (Fam. Umbelliferae)	8.93
*Fa ban xia*	Dried tuber of *Pinelliae ternate (Thunb.)* Breit. (Fam. Araceae)	8.04
*Chuan xiong*	Dried rhizome of *Ligusticum chuanxiong* Hort. (Fam. Umbelliferae)	8.04
*Huai niu xi*	Dried root of *Achyranthes bidentata* Bl. (Fam. Amaranthaceae)	8.04
*Chen pi*	Dried pericarp of the ripe fruit of *Citrus reticulata* Blanco. (Fam. Rutaceae)	5.36
*Sheng gan cao*	Dried root and rhizome of *Glycyrrhiza uralensis* Fisch. (Fam. Leguminosae)	4.46

**Table 2:** Patient characteristics at baseline.

Parameter	TCM group	Control group
	(*n* = 22)	(*n* = 25)
Age (years)	64.82 ± 8.88	60.88 ± 9.41
Gender (men/women)	14/8	17/8
Disease duration (years)	5.44 ± 5.26	6.37 ± 4.93
Duration of levodopa treatment (years)	5.36 ± 5.27	6.12 ± 4.89
Total levodopa daily	405.68 ± 295.80	498.00 ± 313.74
dose (mg)		
Baseline scores		
H&Y score	2.68 ± 1.09	2.24 ± 0.88
PDQ-39 Single Index	28.96 ± 13.19	29.21 ± 15.44
UPDRS Total	55.45 ± 21.48	48.80 ± 18.92
GDS score	16.18 ± 6.79	15.64 ± 6.28
Daily off time (%)	16.65 ± 17.59	22.94 ± 19.24

Values given as mean ± SD.

**Table 3:** Communications, complications of therapy (within group comparisons).

Time	Parameter	TCM group (*n* = 22)	*P*-value	Control group (*n* = 25)	*P*-value
Baseline	PDQ-39 Communications	28.03 ± 23.65		23.75 ± 19.77	
12 weeks		23.86 ± 19.12	.316	30.67 ± 21.07	.015^a^
24 weeks		24.24 ± 19.91	.352	29.67 ± 19.85	.043^a^
Baseline	UPDRS IV	4.22 ± 2.69		5.08 ± 3.48	
12 weeks		3.36 ± 2.42	.013^a^	5.08 ± 3.53	1.000
24 weeks		3.22 ± 2.35	.018^a^	5.12 ± 3.95	.910
Baseline	UPDRS IVC	1.09 ± 0.75		0.76 ± 0.83	
12 weeks		0.64 ± 0.66	.013^a^	0.72 ± 0.74	.755
24 weeks		0.50 ± 0.60	.003^a^	0.68 ± 0.85	.617

^a^Comparison with baseline were statistically significant at *P* < .05.

**Table 4:** Communications, complications of therapy (between group comparisons).

Time (weeks)	Parameter	TCM group (*n* = 22)	Control group (*n* = 25)	*P*-value
12	PDQ-39 Communications	−4.17 ± 19.03	6.92 ± 13.27	.024^a^
24		−3.79 ± 18.67	5.92 ± 13.87	.047^a^
12	UPDRS IV	−0.86 ± 1.49	0.00 ± 0.04	.029^a^
24		−1.00 ± 1.83	1.44 ± 1.74	.085
12	UPDRS IVC	−0.45 ± 0.74	−0.04 ± 0.79	.039^a^
24		−0.59 ± 0.80	−0.08 ± 0.81	.034^a^

^a^Comparison with baseline were statistically significant at *P* < .05.
